# IGOB131, a novel seed extract of the West African plant *Irvingia gabonensis*, significantly reduces body weight and improves metabolic parameters in overweight humans in a randomized double-blind placebo controlled investigation

**DOI:** 10.1186/1476-511X-8-7

**Published:** 2009-03-02

**Authors:** Judith L Ngondi, Blanche C Etoundi, Christine B Nyangono, Carl MF Mbofung, Julius E Oben

**Affiliations:** 1 Laboratory of Nutrition and Nutritional Biochemistry, Faculty of Science, University of Yaounde I, Yaounde, Cameroon; 2 Department of Food Science and Nutrition, ENSAI, University of Ngaoundere, BP 686 Ngaoundere, Cameroon

## Abstract

**Background:**

A recent in vitro study indicates that IGOB131, a novel seed extract of the traditional West African food plant *Irvingia gabonensis*, favorably impacts adipogenesis through a variety of critical metabolic pathways including PPAR gamma, leptin, adiponectin, and glycerol-3 phosphate dehydrogenase. This study was therefore aimed at evaluating the effects of IGOB131, an extract of *Irvingia gabonensis*, on body weight and associated metabolic parameters in overweight human volunteers.

**Methods:**

The study participants comprised of 102 healthy, overweight and/or obese volunteers (defined as BMI > 25 kg/m^2^) randomly divided into two groups. The groups received on a daily basis, either 150 mg of IGOB131 or matching placebo in a double blinded fashion, 30–60 minutes before lunch and dinner. At baseline, 4, 8 and 10 weeks of the study, subjects were evaluated for changes in anthropometrics and metabolic parameters to include fasting lipids, blood glucose, C-reactive protein, adiponectin, and leptin.

**Results:**

Significant improvements in body weight, body fat, and waist circumference as well as plasma total cholesterol, LDL cholesterol, blood glucose, C-reactive protein, adiponectin and leptin levels were observed in the IGOB131 group compared with the placebo group.

**Conclusion:**

*Irvingia gabonensis *administered 150 mg twice daily before meals to overweight and/or obese human volunteers favorably impacts body weight and a variety of parameters characteristic of the metabolic syndrome. This is the first double blind randomized placebo controlled clinical trial regarding the anti-obesity and lipid profile modulating effects of an *Irvingia gabonensis *extract. The positive clinical results, together with our previously published mechanisms of gene expression modulation related to key metabolic pathways in lipid metabolism, provide impetus for much larger clinical studies. *Irvingia gabonensis *extract may prove to be a useful tool in dealing with the emerging global epidemics of obesity, hyperlipidemia, insulin resistance, and their co-morbid conditions.

**Trial registration:**

ClinicalTrials.gov NCT00645775

## Background

Excess adipose tissue increases the workload of the cardiovascular system, adversely alters immune function, and dramatically increases the risk of heart disease, non-insulin-dependent diabetes mellitus, obstructive pulmonary disease, arthritis and a variety of cancers [1]. Although once considered a major health problem isolated to developed countries, obesity is now recognized as a global problem with potentially catastrophic consequences for health economics. In Cameroon, for example, the reported incidence of obesity in urban areas ranges from 17–21% [2]. This dramatic change reflects the social, nutritional, and lifestyle changes involved in rural-urban migration and the urbanization of rural areas. Worthy of note in this context are the changes from traditional high fibre diets and high-activity lifestyles to diets rich in saturated fat and low in fibre, further compounded by reduction in physical activity levels [3,4]. Insulin resistance, caused by the concomitant, complex interaction of environment (e.g. diet, activity level, elevated body mass, excess visceral adiposity) and genetic factors, is a fundamental pathophysiologic cause of the metabolic syndrome, a recognized risk factor for cardiovascular disease [5,6]. Like many other obesity-related conditions, the incidence of the metabolic syndrome is also on the rise in Cameroon [7].

Nutritional management, especially caloric restriction for the purpose of weight reduction, can improve insulin action on target tissues like skeletal muscle, hepatocytes, and peripheral and visceral adipocytes [8,9].

The consumption of *Irvingia gabonensis*, a fleshy West African fruit, is common in traditional Nigerian and Cameroonian cuisine. Initial observations suggested beneficial changes in metabolic parameters were associated with the high fiber content of *Irvingia gabonensis *[10]. Results of subsequent analysis suggested that the earlier observations of the beneficial effects of *Irvingia gabonensis *could not be accounted for by fiber content alone [11]. A recent *in vitro *study using a validated experimental model (murine 3T3-L1 adipocytes) provides compelling data that *Irvingia gabonensis *seed extract may inhibit adipogenesis through modulation of PPAR gamma and glycerol-3 phosphate dehydrogenase in addition to beneficial impact upon leptin and adiponectin [12].

The current clinical study was designed to evaluate if the *in vitro *data could translate into *in vivo *clinical efficacy in overweight and/or obese human volunteers.

## Participants and methods

### Study population

Participants for the study were recruited from the city of Yaoundé, Cameroon and surrounding metro region through radio and print media advertisement. Inclusion criteria for study participation mandated: (1) men and women ages 19 to 50 years, in general good health free from significant medical illness that, in the opinion of the investigator, could adversely compromise study participation for any reason; (2) stable body weight (+/- 2 kg) for at least three months prior to study randomization without use of medication known or suspected to affect body weight or appetite; (3) no concomitant or recent (within the past three months) bacterial or seasonal viral infection (e.g. influenza); (4) BMI between 26 kg/m^2 ^and 40 kg/m^2^; (5) no attempts at weight loss through dietary intervention over the three months prior to trial randomization; (6) no participation in a structured weight control program for at least three months prior to study randomization; (7) non-smoker; (8) non-drinkers of sugary fizzy drinks; (9) capability and willingness for multiple blood sampling procedures; and (10) ability to competently understand and sign the consent form.

Based on the above criteria, 120 consenting volunteers (62 males and 58 females) were selected to participate in the study. The protocol used was approved by the Cameroon National Ethics Committee; the study was conducted in accord with the Helsinki Declaration (1983 version).

### Study design/intervention

The study was a randomized, double-blind, placebo-controlled design for 10 weeks. The volunteers were randomly divided into two groups – placebo or IGOB131. The participants consumed either one capsule of placebo or one capsule of IGOB131 (containing 150 mg of *Irvingia gabonensis *extract) 30–60 minutes before lunch and dinner (BID) throughout the study period.

### Test materials

All test materials were supplied by Gateway Health Alliances, Inc. (Fairfield, CA, USA) in individual packets of capsules. The identical-looking placebo and active formulation capsules contained, respectively, maize-based powder consisting of 150 mg maltodextrin, or 150 mg IGOB131.

### Anthropometric measurements

Height was measured with a locally manufactured wall-mounted stadiometer, which was calibrated against the Cameroon's Department of National Security identification scale. Body weight and percent body fat, were assessed using a Tanita™ BC-418 Segmental Body Composition Analyzer/Scale that uses bio-electrical impedance analysis for body composition analysis. Body mass index (BMI) was calculated using the weight and height measurements. Waist circumference measurements to the nearest 0.1 cm were taken at the mid-point between the bottom rib and the hip bone, without restrictive garments using a flexible non expandable tape measure.

### Dietary Intake

Three days before the collection of baseline measurements and three times during the trial, dietary intake of subjects was evaluated by a qualified dietician. Energy and protein intakes were calculated using standard food composition tables based upon the 3-day food intake record.

No major dietary intervention or formal physical activity program was instituted during the course of the study; participants were instructed to maintain the current level of physical activity prior to study randomization during the course of the study.

### Sample collection

Fasting blood samples (5 ml of blood) were collected at baseline, and at 4, 8, and 10 weeks. Serum obtained from each blood sample was split into multiple 500 μl aliquots and stored at -20°C until needed for the measurement of total cholesterol, LDL cholesterol, fasting blood glucose, C-reactive protein, adiponectin, and leptin levels.

### Analytical methods

Determination of glucose in blood using glucose oxidase with an alternative oxygen receptor was adopted for this study [13]. Serum total cholesterol was assayed by the cholesterol oxidase method [14] while triglycerides as well as serum glucose levels were assayed following the method described by Buccolo & David [15]. Quantitative determinations of C-reactive protein (CRP) was assessed using a high sensitivity immunoassay (Oxis International, Foster City, CA. USA) while serum leptin was determined in duplicate using an enzyme-linked immunosorbent assay (ELISA) (Diagnostic Systems Laboratory, Webster, TX. USA) technique within the same assay, with an intra-assay variance of 3.2%. Serum adiponectin was measured in duplicate using an enzyme immunoassay (APLCO Diagnostics, Salem, NH. USA) within the same assay. Intra-assay variance was ≤ 5%.

### Statistical analysis

Statistical analyses (Student's t-test and ANOVA) were performed using the Statistical Package for the Social Sciences (SPSS) software. Graphical representations of relative changes in measured variables were performed using Sigma Plot statistical package.

## Results

The 120 volunteers (mean age 34, range 19–50) were divided into 2 groups, with 60 randomly assigned to placebo and 60 to IGOB131 (active). Of these initial 120 volunteers, 102 completed the 10 week study; 50 from the placebo group, and 52 from the IGOB131 group. Eighteen volunteers [placebo (n = 12) and IGOB131 (n = 6)] dropped out of the study for the following reasons – not experiencing rapid weight loss (n = 10); influenza attack (n = 3); dryness of mouth (n = 3); no reason given (n = 2).

### Food Intake

The mean daily energy intake in the IGOB131 group was 2767 ± 187 kcal of which 56% was from carbohydrate, 29% from protein, and 15% from fat. In the placebo group, the mean daily energy intake was 3156 ± 185 kcal of which 56% was from carbohydrate, 29% was from protein, and 15% was from fat. Seven-day dietary and activity assessment of the subjects at baseline showed similar food intake habits and energy levels.

#### Body weight, waist size and body fat

Baseline characteristics of the two groups were well-matched and without significant differences at baseline (Table 1). There were no significant differences in the baseline body weight, waist circumference and serum leptin measurements between the placebo and experimental group (Table 1). However, by the tenth week, significant differences were observed between the placebo and experimental intervention groups, respectively, for body weight (95.7 kg vs. 85.1 kg, respectively, p < 0.01), waist circumference (101.1 cm vs. 88.1 cm, respectively, p < 0.05). Body fat decreased over time in both groups but the experimental group lost significantly more body fat (6.3%, p < 0.05) compared to the placebo group (1.9%) (Table 1).

**Table 1:** Changes* in body weight, waist, leptin, fat, total cholesterol, LDL cholesterol, glucose and adiponectin measurements in the course of the study.

			**Time (weeks)**			
**Variables**	**Group**	**Base line**	**4**	**8**	**10**	**Over change**

Weight (Kg)	Placebo	96.4 ± 12.3	95.8 ± 8.2	95.1 ± 10.6	95.7 ± 15.2	-0.7
	IGOB131	97.9 ± 9.1	94.3 ± 5.5 ^a^	89.7 ± 4.7 ^a^	85.1 ± 3.1^b^	-12.8
						
Waist (cm)	Placebo	106.4 ± 10.8	102.7 ± 10.6	101.1 ± 14.8	101.1 ± 15.8^b^	-5.3
	IGOB131	105.1 ± 6.3	98.0 ± 8.4^a^	90.1 ± 8.70^a^	88.1 ± 7.6	-16.19
						
Leptin (ng/ml)	Placebo	31.3 ± 1.8	29.4 ± 1.4	28.1 ± 1.8	28.4 ± 1.8	-2.9
	IGOB131	32.9 ± 1.6	18.1 ± 1.3^a^	16.8 ± 1.2^a^	16.9 ± 1.3^b^	-16.0
						
Fat (%)	Placebo	34.7 ± 8.6	33.31 ± 10.9	32.9 ± 11.9	32.7 ± 15.7	-1.99
	IGOB131	34.2 ± 7.9	31.6 ± 5.4^a^	28.2 ± 6.6^a^	27.9 ± 5.5^a^	-6.3
						
LDL cholesterol (mg/dL)	Placebo	77.4 ± 9.2	76.5 ± 8.9	74.1 ± 9.3	73.7 ± 8.3	-3.75
	IGOB131	82.2 ± 8.1	71.8 ± 5.9^b^	64.7 ± 8.9^b^	59.8 ± 5.0^b^	-22.44
						
Total cholesterol (mg/dL)	Placebo	145.2 ± 22.4	143.9 ± 12.2	142.1 ± 13.6	142.4 ± 12.1	-2.8
	IGOB131	151.7 ± 18.5	133.7 ± 16.6^a^	120.1 ± 11.9 ^a^	111.9 ± 5.8^a^	-39.8
						
C-reactive protein (mg/L)	Placebo	1.46 ± 0.05	1.46 ± 0.04	1.445 ± 0.03	1.455 ± 0.05	-0.01
	IGOB131	1.49 ± 0.041	0.91 ± 0.05^a^	0.71 ± 0.04^b^	0.72 ± 0.05^b^	-0.78
						
Glucose (mg/dL)	Placebo	81.4 ± 9.6	79.5 ± 10.1	77.8 ± 9.4	77.1 ± 7.8	-4.3
	IGOB131	85.55 ± 5.59	76.6 ± 10.3^a^	68.7 ± 9.3^a^	66.3 ± 4.9^a^	-19.3
						
Adiponectin (mg/L)	Placebo	12.1 ± 3.2	15.2 ± 3.5	14.6 ± 3.5	14.94 ± 3.9	+ 2.8
	IGOB131	12.2 ± 3.0	24.8 ± 3.8^a^	31.0 ± 4.0^b^	31.6 ± 3.9^a^	+ 19.4

^a ^P < 0.05; ^b ^P < 0.01 compared with Placebo

**Figure 1:**
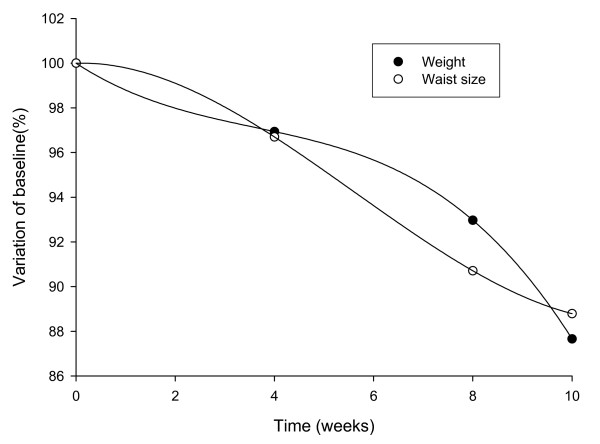
**Changes in body weight and waist circumference measurements in IGOB131 group corrected for placebo values**.

When corrected for placebo response, the pattern of relative changes in weight and waist circumference was found to be different between the two groups, consistent with a difference in response to the intake of the extract (Figure 1).

#### Total and LDL cholesterol, C-reactive proteins, leptin

These variables decreased from baseline, though at different rates and magnitudes associated with duration of the study, with the experimental group showing statistically significant changes compared with the placebo group at week-10 (Table 1).

**Figure 2:**
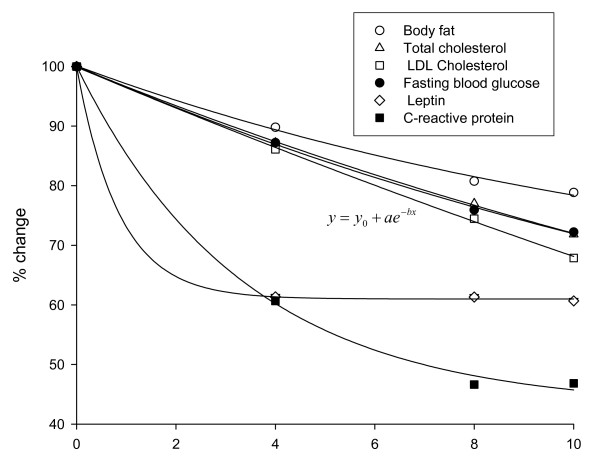
**Changes in measured characteristics in IGOB131 group corrected for placebo values**.

While baseline levels of serum lipids were similar in the two groups, significant differences were observed between the two as the study progressed with the experimental group showing progressively greater improvement. At week-10, significant differences were observed for total cholesterol (placebo: 142.5 mg/dl vs. IGOB131: 111.9 mg/dl, p < 0.05) and LDL cholesterol (placebo: 77.7 mg/dl vs IGO131: 59.77 mg/dl, p < 0.01). Compared to baseline values, total cholesterol decreased by 1.9% in the placebo group as opposed to 26.2% for the IGOB131 group while LDL cholesterol levels fell by 4.8% in the placebo compared to 27.3% in the IGOB131 group. Correcting for placebo values, the relative change in the total cholesterol and LDL cholesterol was observed to follow a similar pattern (Figure 2) in the experimental group, suggesting a similar response mechanism to IGOB131 intake.

Like in the case of the other parameters measured, C-reactive protein and leptin levels decreased over time in both groups. The rate of decrease was however accelerated in the IGOB131 relative to the placebo-group. Serum levels of CRP fell by 1.2% in the placebo group as opposed to 52.0% in the experimental group relative to baseline. On the other hand, leptin levels decreased by 9.3% in the placebo group compared to a 48.6% in the IGOB131 group over the 10-week experimental period.

### Fasting blood glucose levels

**Figure 3:**
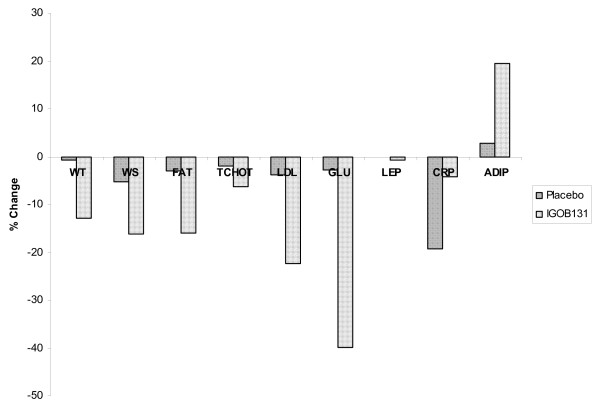
**Percentage decrease in body weight (WT), Waist size (WS), Fat (FAT), Total Cholesterol (TCHOL), LDL cholesterol (LDL), Glucose (GLU), Leptin (LEP), C-reactive protein (CRP) and Adiponectin (ADIP) after 10 weeks of use of extract IGO131**.

Blood glucose levels in the experimental group (85.6 mg/dl ± 5.6 mg/dl) and in the placebo group (81.4 ± 9.6 mg/dl) were similar at baseline but decreased to significantly different levels (P < 0.05) at week-10 of the study (Table 1). In relative terms, decreases in placebo and treatment groups were 5.3% vs. 22.5%, respectively (Figure 3). Corrected for the placebo values, the changes in blood glucose levels were similar to that of lipids (Figure 2).

### Adiponectin levels

**Figure 4:**
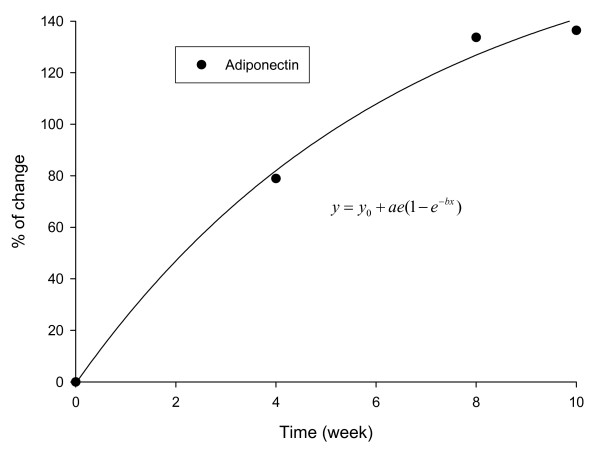
**Changes in the adiponectin levels in IGOB131 group corrected for placebo**.

Baseline serum adiponectin levels were comparable between the two groups (placebo: 12.1 mg/l ± 3.21 mg/l; IGOB131: 12.16 mg/l ± 3.04 mg/l), but increased with time (Table 1). By week-10 of the study adiponectin levels in the placebo group increased by 23.4% compared to 159.8% in the experimental group. Corrected for placebo values, the rate of increase in serum adiponectin levels in IGOB131 group followed an exponential curve (Figure 4).

### Adverse events

Treatment of overweight and obese subjects with IGOB131 was well-tolerated. Adverse events with an incidence >n = 3 included headache (n = 5), sleep difficulty (n = 6), and intestinal flatulence (n = 6). The incidence of all reported side effects was similar in the placebo group as well as in the treatment group.

## Discussion

The present study suggests that IGOB131, a seed extract of *Irvingia gabonensis*, safely and significantly reduces body weight in overweight and/or obese subjects, and has a favorable impact upon a variety of other metabolic parameters. The current clinical study shows IGOB131 administration is associated with increases in plasma adiponectin levels and decreases in leptin and CRP levels in comparison with the placebo group. These observations support the results of a recent *in vitro *study [12] which suggests a favorable impact upon adipogenesis associated with IGOB131.

Insulin resistance is the hallmark of the metabolic syndrome and is strongly associated with excess adiposity [16]. A variety of adipocyte-derived biologically active molecules have been identified, including leptin, resistin, TNF-α, and IL-6, that may contribute to obesity-linked metabolic abnormalities [17]. Since plasma leptin levels are closely correlated with the level of adipose tissue [18], the decreases in plasma leptin level associated with IGOB131 treatment may be attributable to the decrease of adipose tissue induced as a consequence of weight loss.

According to McTernan et al. [17], adipose tissue plays a prominent role in the clinical expression of metabolic syndrome, most likely mediated by the increased release and peripheral tissue action of non-esterified fatty acids and by the dysregulated production of adipocyte-secreted proteins, including leptin, adiponectin, resistin, TNF-α, and IL-6. Adiponectin, known to correlate with endothelial function and vascular health, is exclusively expressed in adipose tissue and abundant in human plasma, and appears to be decreased in individuals with obesity and type 2 diabetes, risk factors for atherosclerosis[19].

The observation of a significant weight loss, as well as an increase in adiponectin is consistent with earlier reports in the literature observing an association between weight loss and an increase in adiponectin levels [20].

IGOB131 is relatively rich in plant-derived protein and antioxidants. A study by Baum et al, [21] had earlier shown a reduction of both total cholesterol and triglycerides levels in a hypercholesterolemic man who was fed a plant protein diet. Other studies have shown metabolic syndrome-preventive activity of antioxidant components (e.g. vitamin C, polyphenols). A number of polyphenols such as epigallocatechin gallate have anti-obesity activity and may improve metabolic disorders via modulation of adipokines and growth factors, including metabolic improvement of leptin function [22-24]. Although the active principles of IGOB131 have not yet been fully identified, it is possible that it contains some of the above mentioned compounds.

In conclusion, *Irvingia gabonensis *extract administered twice a day to healthy, overweight and obese individuals resulted in both weight reduction (body weight, body fat, waist size) and an improvement in metabolic parameters associated with insulin resistance. The current results suggest that IGOB131 may be a helpful adjunct in the management of overweight and/or obesity, supporting previous suggestions from our laboratory [25].

## Competing interests

Part of the funds for carrying out this study was provided by Gateway Health Alliances Inc., through their "Encouragement of Research in developing countries Initiative".

## Authors' contributions

JEO conceived, designed and coordinated the work, as well as prepared the manuscript; CMFM was involved in the co-design of the work as well as the draft of the manuscript. JLN carried out analytical work, BCE carried out analytical work and contributed in drafting the manuscript; CBN carried out analytical and statistical analysis. All authors read and approved the final manuscript.
